# High molecular weight hyaluronan protects cartilage from degradation by inhibiting aggrecanase expression

**DOI:** 10.1002/jor.24126

**Published:** 2018-09-07

**Authors:** Takashi Ohtsuki, Keiichi Asano, Junko Inagaki, Akira Shinaoka, Kanae Kumagishi‐Shinaoka, Mehmet Z. Cilek, Omer F. Hatipoglu, Toshitaka Oohashi, Keiichiro Nishida, Issei Komatsubara, Satoshi Hirohata

**Affiliations:** ^1^ Department of Medical Technology, Graduate School of Health Sciences Okayama University 2‐5‐1, Shikata‐cho Okayama Japan; ^2^ Department of Molecular Biology and Biochemistry, Okayama University Graduate School of Medicine Dentistry and Pharmaceutical Sciences 2‐5‐1, Shikata‐cho Okayama Japan; ^3^ Department of Cell Chemistry, Okayama University Graduate School of Medicine Dentistry and Pharmaceutical Sciences 2‐5‐1, Shikata‐cho Okayama Japan; ^4^ Department of Human Morphology, Okayama University Graduate School of Medicine Dentistry and Pharmaceutical Sciences 2‐5‐1, Shikata‐cho Okayama Japan; ^5^ Department of General Internal Medicine I, Kawasaki Hospital Kawasaki Medical School 2‐1‐80, Nakasange, Kita‐ku Okayama Japan

**Keywords:** aggrecan, chondrocyte, hyaluronic acid, metalloproteinase

## Abstract

Hyaluronan (HA) is an extracellular matrix (ECM) component of articular cartilage and has been used to treat patients with osteoarthritis (OA). A disintegrin and metalloproteinases with thrombospondin motifs (ADAMTSs) play an important role in cartilage degradation in OA. We have previously reported that ADAMTS4 and ADAMTS9 were induced by cytokine stimulation. However, the effect of HA on the cytokine‐inducible ADAMTS9 has never been investigated. Moreover, it is unclear whether HA protects cartilage by suppressing aggrecan degradation. Here, we examined the effects of HA on ADAMTS expression in vitro and on cartilage degradation in vivo. ADAMTS9 expression was higher than that of the other aggrecanases (ADAMTS4 and 5) in human chondrocytes, chondrocytic cells, and rat cartilage. *ADAMTS4* and *9* mRNA levels were upregulated in cytokine‐stimulated chondrocytes and chondrocytic cells. Pre‐incubation with HA significantly inhibited *ADAMTS9* mRNA expression in cytokine‐stimulated cells. In a rat OA model, *Adamts5* and *9* mRNA levels were transiently increased after surgery; intra‐articular HA injections attenuated the induction of *Adamts5* and *9* mRNA. HA also blocked aggrecan cleavage by aggrecanase in OA rats in a molecular size‐dependent manner. These results demonstrate that HA attenuates induced aggrecanases expression in OA and thereby protects articular cartilage degradation by this enzyme. Our findings provide insight into the molecular basis for the beneficial effects of HA in OA. © 2018 The Authors. *Journal of Orthopaedic Research®* Published by Wiley Periodicals, Inc. on behalf of Orthopaedic Research Society. J Orthop Res 36:3247–3255, 2018.

Osteoarthritis (OA) is the most common type of cartilage disease and results from the degradation of extracellular matrix (ECM) in the joints. Articular cartilage consists of chondrocytes and ECM molecules including aggrecan, type II, IX, and XI collagen, and hyaluronan (HA).^1^ Aggrecan holds water in the matrix, allowing cartilage to resist mechanical forces^2^; it binds HA through its G1 domain^3^ as part of a large complex consisting of 10–100 aggrecan molecules per HA.^4^ Aggrecan as well as collagen is degraded in OA cartilage^5^ owing to the activity of matrix metallo proteinases and aggrecanases that target aggrecan cleavage sites.^6^ Aggrecanase 1 and 2 (also known as ADAMTS4 and 5, respectively) cleave aggrecan at Glu373–Ala374 in bovine nasal cartilage stimulated with interleukin (IL)‐1.^7^, ^8^ Other ADAMTSs (e.g., ADAMTS1 and 9) also cleave aggrecan at this site.^9^, ^10^

HA is a linear polysaccharide composed of N‐acetylglucosamine and glucuronic acid that is a major component of the ECM in articular cartilage and synovial fluid.^11^, ^12^ It was observed that HA in the knee synovial fluid of patients with OA reduced in both concentration and molecular weight.^13^ HA was originally administered for OA intra‐articular injection therapy in order to compensate for the reduction in the level of endogenous HA. HA displays its viscosity in a molecular weight‐dependent manner.^14^ In addition to its several biophysical properties (viscosity, water retention, lubrication),^15^, ^16^ intra‐articular HA injection therapy has been devised assuming that HA may have several disease‐modifying effects in OA. However, evidence of its molecular effects has not been clearly established and its mechanisms of action and clinical benefits remain in need of confirmation. A recent study has revealed that HA has biological functions ranging from immune regulation,^17^ wound healing,^18^, ^19^ and induction of differentiation in embryonic cells,^20^ with its effects dependent on molecular size.^21^ Articular joints mainly consists of cartilage, bones, and synovial fluid. In order to analyze OA development and protection by HA in organ and tissue levels, we selected rat OA surgery model.

In this study, we examined the effects of HA on the expression of *ADAMTSs* in cytokine‐stimulated chondrocytes, as well as its protective function in knee cartilage in a rat OA model.

## METHODS

### Reagents

Recombinant human IL‐1β and tumor necrosis factor (TNF)α were purchased from R&D Systems (Minneapolis, MN) and stored at −80°C and diluted in culture medium immediately before use. HA molecules of different sizes—that is, 300, 800, and 2700 kDa (HA300, HA800, and HA2700, respectively)—were used in this study. All HA molecules used in this study were purified from *Streptococcus equi*. HA size was determined using a Multi‐Angle Laser Light Scattering method. Endotoxin concentration was below the level of detection (<0.06 EU/g). HA was kindly supplied by Chugai Pharmaceutical (Tokyo, Japan).

### Cell Culture

Normal human articular chondrocytes (NHAC‐kn) were purchased from Lonza (Walkersville, MD). OUMS‐27 chondrosarcoma cells were prepared as previously described.^22^ Cells were cultured in Dulbecco's modified Eagle's medium containing 10% fetal bovine serum and penicillin/streptomycin at 37°C in a humidified atmosphere of 5% CO_2_. Cells were passaged at ratios of 1:2 to 1:4 by using trypsin with ethylenediaminetetraacetic acid (EDTA) every 7–10 days. The medium was replaced every 3 days. Cells at passages 3–6 were used for all experiments; 2.5 × 10^5^ cells were plated in 6‐well plates for 2 days and the medium was replaced with serum‐free medium for 24 h before cytokine stimulation. Cells were pretreated with HA or left untreated for 5 h, and then cultured in the presence of IL‐1β and TNFα (both at 10 ng/ml).

### Quantitative (qRT)—PCR

Following cytokine stimulation, cells were washed twice with phosphate‐buffered saline (PBS); total RNA was extracted using TRIzol reagent (Invitrogen, Carlsbad, CA) according to the manufacturer's instructions and reverse transcribed as previously described.^23^, ^24^ Briefly, genomic DNA was removed by treatment with 5 U DNase I (Roche Diagnostics, Lewes, UK) at 37°C for 15 min, followed by inactivation at 65°C for 10 min, and 2 µg total RNA were reverse transcribed with random primers (Toyobo, Osaka, Japan). qRT‐PCR was carried out on a StepOnePlus system (Applied Biosystems, Foster City, CA) as previously reported, with slight modification.^25^, ^26^ Briefly, each reaction mixture contained 5 µl TaqMan Fast Advanced Master mix, 0.5 µl TaqMan Gene Expression assay for target genes (ADAMTS4, 5, and 9; aggrecan; and type II collagen) and the endogenous control (glyceraldehyde 3‐phosphate dehydrogenase, GAPDH), and 4 µl cDNA. Cycling conditions were as follows: 95°C for 20 s; and 40 cycles of 95°C for 1 s and 60°C for 20 s. All samples were analyzed in triplicate. TaqMan primers and probes (human ADAMTS4: assay ID Hs00192708_m1 based on RefSeq NM_005099.4; human ADAMTS5: assay ID Hs00199841_m1 based on RefSeq NM_007038.3; human ADAMTS9: assay ID Hs00172025_m1 based on RefSeq NM_182920.1; human MMP‐13: assay ID Hs00233992_m1 based on RefSeq NM_002427.3; human GAPDH: assay ID Hs02758991_g1 based on RefSeq NM_001256799.1 and NM_002046.4; rat ADAMTS4: assay ID Rn02103282_s1 based on RefSeq NM_023959.1; rat ADAMTS5: Rn01458486_m1 based on RefSeq NM_198761.1; rat ADAMTS9: Rn01425216_m1 RefSeq NM_001107877.1; rat GAPDH: Rn01462662_g1 RefSeq NM_017008.4) and TaqMan Fast Advanced Master Mix were purchased from Applied Biosystems. GAPDH was used to normalize the levels of target RNA with the comparative Ct (ΔΔCT) method as previously described.^27^, ^28^, ^29^ Values obtained from untreated cells were considered as controls.

### Rat Model of OA

Male Sprague Dawley rats (*n* = 190, aged 6 weeks) were used in this study (Japan SLC, Shizuoka, Japan). Rats were housed in standard cages (2 rats/cage) in a temperature‐controlled room (20–25°C), with 12‐h light/dark cycles. Standard rodent chow and water were provided ad libitum. All protocols involving experimental animals conformed to the local institutional guidelines for animal care, which are based on the Guide for the Care and Use of Laboratory Animals (National Institutes of Health publication no. 85–23, revised 1996). Rats were randomly divided into control and procedure groups. Animals were anesthetized by intraperitoneal injection of sodium pentobarbital (50 mg/kg). An incision was made to the right knee joint, and arthrotomy was performed using a medial parapatellar approach.^30^ The anterior cruciate and medial collateral ligaments were transected with surgical scissors. The medial joint space was opened by applying a valgus force. Two‐thirds of the anterior medial meniscus at the posterior corner were resected using surgical scissors. For sham surgery (control animals), an incision was made to the right knee joint. Total RNA was isolated from rat femoral cartilage using TRIzol reagent and grinding excised cartilage in liquid nitrogen using a ceramic mortar and pestle.

### Histological Analysis

Full thickness sagittal sections of cartilage in the weight‐bearing area of the medial femoral condyle were examined. Knee joint samples were dissected, fixed in 4% paraformaldehyde for 24 h, and decalcified in 0.3 M EDTA (pH 7.5) for 14 days before embedding in paraffin. Sections (5‐µm‐thick) were stained with 0.1% Safranin O. The severity of OA lesions was classified by two independent researchers according to the Osteoarthritis Research Society International (OARSI) cartilage assessment system, which provides a semi‐quantitative method of evaluating the histopathology of OA cartilage with an index combining grade and stage (0–24 points).^31^

### Intra‐Articular Injection of HA

Animals were divided into five groups (*n* = 10 each): sham, injected with PBS, or injected with HA300, 800, or 2700. For histological analysis or evaluation of aggrecan degradation, rats in the treatment groups were anesthetized with diethyl ether and injected with HA (50 µl of 10 mg/ml) or 0.01 M PBS twice a week in the right knee joint from the medial side of the patellar tendon with the knee joint flexed to 90°. We confirmed that the tip of the needle reached the joint space by lack of resistance or aspiration of joint fluid. Animals were sacrificed 6 weeks after surgery, and right knee joint samples were obtained at the indicated times.

### Immunohistochemistry

Aggrecan cleavage was assessed by immunohistochemistry in knee joint sections using neoepitope antibody (anti‐NITEGE), which recognizes the aggrecan Glu373‐Ala374 cleavage site. Deparaffinized sections were pre‐treated with chondroitinase ABC (1 U/ml) (Sigma, St. Louis, MO) at 37°C for 2 h as previously described. Endogenous peroxidase was blocked with 3% H_2_O_2_ in PBS at room temperature for 15 min, and sections were incubated in normal goat serum at room temperature for 60 min. Rabbit anti‐NITEGE antibody (10 μg/ml) (Affinity BioReagents, Golden, CO) was applied overnight at 4°C, with sections treated with PBS instead of primary antibody serving as a negative control. Histofine Simple Stain Rat MAX PO(R) (Nichirei, Tokyo, Japan) was used as the secondary antibody. The reaction was visualized with Histofine Simple Stain Diaminobenzidine (Nichirei). Sections were counter stained with hematoxylin.

### Statistical Analysis

Data are expressed as the mean ± S.D. For multiple comparisons, analysis of variance (ANOVA) was performed and post‐hoc analysis with Bonferroni's test was employed. The Tukey–Kramer method was used to evaluate differences in OARSI scores. *p* values <0.05 were considered statistically significant.

## RESULTS

### Inflammatory Cytokines Induce ADAMTS Expression in Chondrocytes

#### ADAMTS mRNA Expression in OUMS‐27 Cells

**Figure 1:**
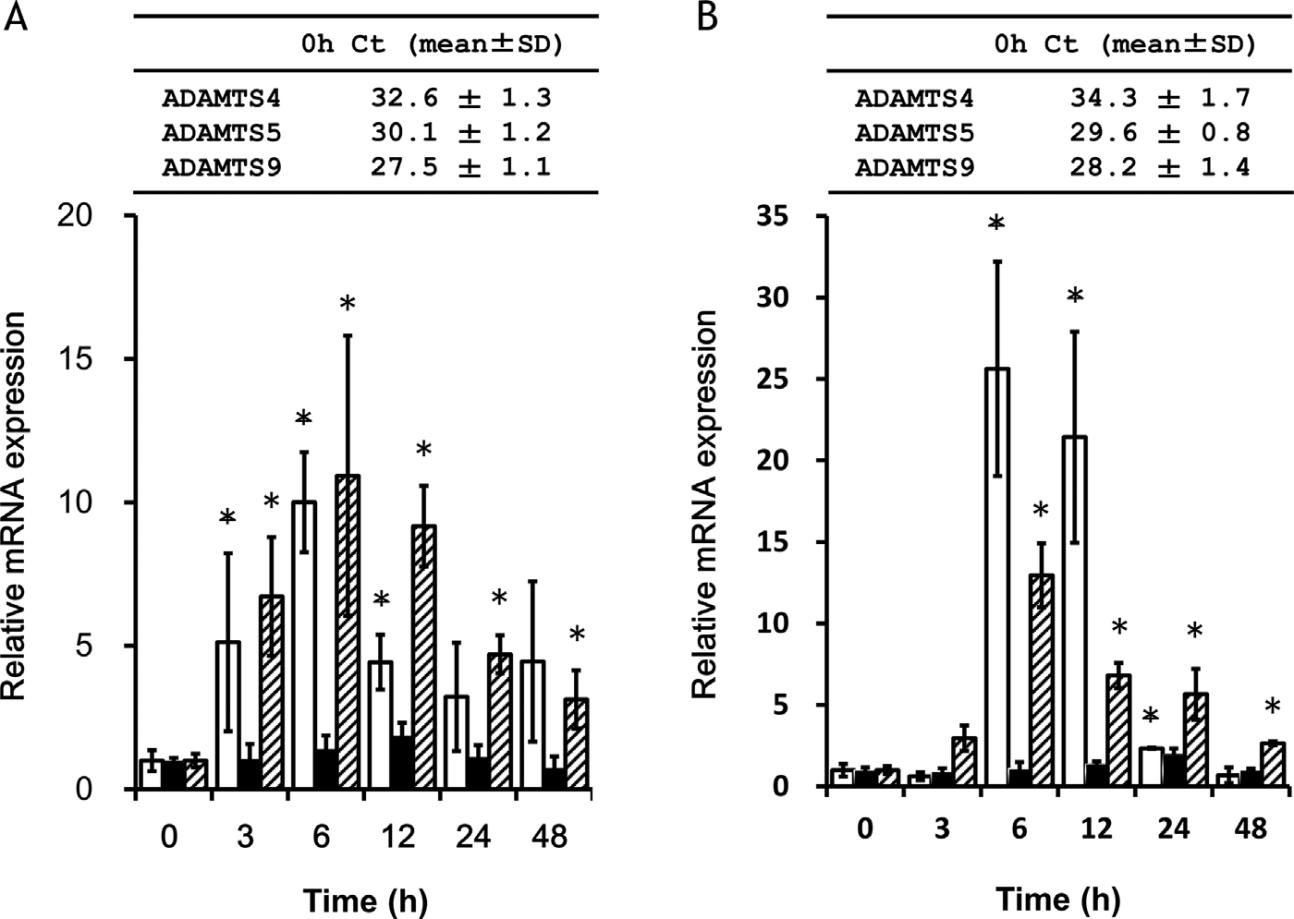
Cytokine‐induced ADAMTS expression in chondrocytes and chondrocytic cells. (A) NHAC‐kn and (B) OUMS‐27 cells were stimulated for 0–48 h with 10 ng/ml IL‐1β and TNFα. *ADAMTS4*, *5*, and *9* transcript levels were evaluated relative to levels in unstimulated cells by qRT‐PCR. Basal expression of ADAMTS4, 5, and 9 mRNA determined by qRT‐PCT were represented as threshold cycles (Ct) on the top of graph. Values represent mean ± S.D. (*n* = 6 per group). Open bars, ADAMTS4; solid bars, ADAMTS5; hatched bars, ADAMTS9. *p < 0.05 versus control.

We examined whether inflammatory cytokines induce mRNA expression of *ADAMTS4*, *5*, and *9* in NHAC‐kn and OUMS‐27 cells by qRT‐PCR. Co‐treatment with IL‐1β and TNFα increased the mRNA levels of *ADAMTS4* and *9* but not *ADAMTS5* in NHAC‐kn (Fig. 1A) and OUMS‐27 (Fig. 1B) cells, with levels peaked at 6 h after the application. The baseline Ct values of ADAMTS4, 5, and *9* were 32.6, 30.1, and 27.5, respectively, in NHAC‐kn cells. The baseline Ct values of ADAMTS4, 5, and *9* were 34.3, 29.6, and 28.2, respectively, in OUMS‐27 cells. The kinetic change of each *ADAMTS* mRNA by cytokine stimulation was almost similar in these two cell lines (NHAC‐kn and OUMS‐27).

### HA Attenuates Inflammatory Cytokine‐Induced *ADAMTSs/MMP* mRNA Expression

**Figure 2:**
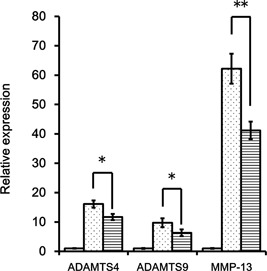
HA suppresses *ADAMTS/MMP* mRNA expression in cytokine‐stimulated chondrocytic cells. OUMS‐27 cells were pretreated with or without 1.0 mg/ml HA2700 for 5 h, then stimulated with 10 ng/ml IL‐1β and TNFα for 6 h. Open bars; nontreatment, dotted bars; cytokine stimulation for 6 h without HA pretreatment, horizontal bars; HA pretreatment; and cytokine stimulation for 6 h. HA pretreatment of OUMS‐27 significantly attenuated *ADAMTS4*, *ADAMTS9*, and *MMP‐13* mRNA expression induced by cytokines. The decline ratio was 27%, 27%, 35%, and 34% of *ADAMTS4* mRNA, *ADAMTS9* mRNA, and *MMP‐13* mRNA respectively.

We next assessed the effect of HA of different sizes on cytokine‐induced *ADAMTS9* mRNA expression. Pretreatment with HA of 800 and 2,700 kDa significantly cytokine‐induced increase in *ADAMTS9* expression in OUMS27 cells; this effect was especially remarkable for the largest HA molecule (i.e., HA2700) (Fig. S1‐A). Cytokine‐induced *ADAMTS9* mRNA expression also attenuated in NHAC‐kn by HA2700 (Fig. S‐1B). In cells pretreated with various concentrations of HA 2700 for 5 h and then stimulated with IL‐1β and TNFα for 6 h, the cytokine‐induced upregulation of *ADAMTS9* expression was significantly attenuated at 1,000 µg/ml HA2700 (Fig. S‐1C). These results demonstrate that HA2700 significantly inhibits the induction of ADAMTS9 expression by cytokines in a concentration‐ and molecular size‐dependent manner. Furthermore, cytokine‐induced ADAMTS4 and MMP‐13 mRNA expressions were also attenuated by HA2700 (Fig. 2). HA removal after 5 h HA pretreatment did not attenuate cytokine‐induced ADAMTS9 mRNA expression (Fig. S‐2). Five hours of HA treatment alone did not influence ADAMTS9 mRNA expression (Fig. S‐3).

### HA Recovered Inflammatory Cytokine‐Reduced Expression of ECM Components

We examined whether inflammatory cytokines alter the expression of the cartilage ECM components aggrecan and type II collagen in OUMS‐27 cells by qRT‐PCR. When OUMS‐27 cells were stimulated with IL‐1β and TNFα, both *aggrecan* and *type II collagen* mRNA expression decreased in a time‐dependent manner (Figs. S‐4A and 4B). When NHAC‐kn cells were stimulated with IL‐1β and TNFα, both *aggrecan* and *type II collagen* mRNA expression decreased in a time‐dependent manner (Figs. S‐4C and 4D). Both cell lines reacted in a same dependency. To determine whether HA influences cytokine‐induced changes in *aggrecan* and *type II collagen* mRNA expressions, cells were pretreated with HA for 5 h and then stimulated with IL‐1β and TNFα for an additional 24 h; transcript levels were then examined. HA800 and HA2700, but not HA300, restored the expression of *aggrecan* and *type II collagen* mRNA that was decreased by cytokine treatment in OUMS‐27 cells (Figs. S‐4E and 4F). HA2700 pretreatment itself up‐regulated aggrecan and type II collagen mRNA (Figs. S‐4E and 4F).

### *Adamts* Expression Is Upregulated in a Rat Model of OA

**Figure 3:**
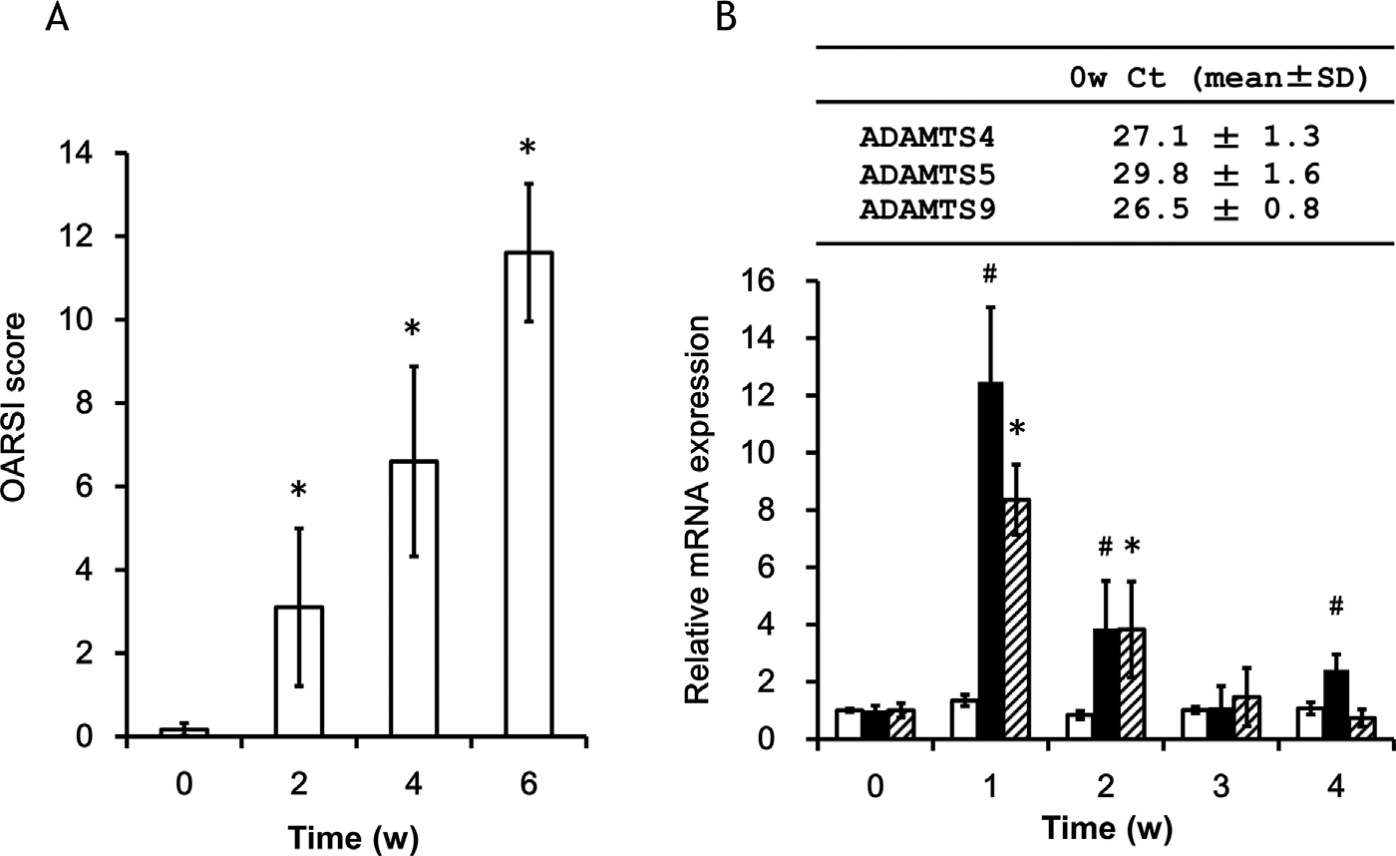
OARSI score and *ADAMTS* mRNA expression in a rat OA model. (A) Histological analysis of medial femoral condyle cartilage of OA rats assigned an OARSI score after surgery (*n* = 10 per group). *p < 0.05 versus sham‐operated rats. (B) *ADAMTS4*, *5*, and *9* mRNA expression was determined by qRT‐PCR (*n* = 10 per group). Basal expression of ADAMTS4, 5, and 9 mRNA determined by qRT‐PCR were represented as threshold cycles (Ct) on the top of graph. Total RNA was extracted 0, 1, 2, 3, and 4 week(s) after knee surgery. Open bars; ADAMTS4, closed bars; ADAMTS5, hatched bars; ADAMTS9. Levels of *ADAMTS5* and *9* mRNA were upregulated in OA rats. * and ^#^ indicate *p* < 0.05 versus sham‐operated rats.

Histological changes in articular cartilage in rat OA model were assessed 2, 4, and 6 weeks after surgery by Safranin O staining. Cartilage destruction was evaluated by using OARSI scores, which increased after surgery (Fig. 3A) but showed little change for those of the sham surgery group (data not shown), confirming the validity of our rat OA model.

The mRNA expression of *Adamts* was assessed by qRT‐PCR in our rat OA model 1, 2, 3, and 4 weeks after surgery. *Adamt5* and *9* transcripts were upregulated to maximum levels 1 week post‐surgery before gradually declining thereafter (Fig. 3B). The baseline Ct values of ADAMTS4, 5, and *9* were 27.1, 29.8, and 26.5, respectively, in Rat OA model (Fig. 3B).

### HA Inhibits the Increase in *Adamts* mRNA Expression Induced by OA Model

**Figure 4:**
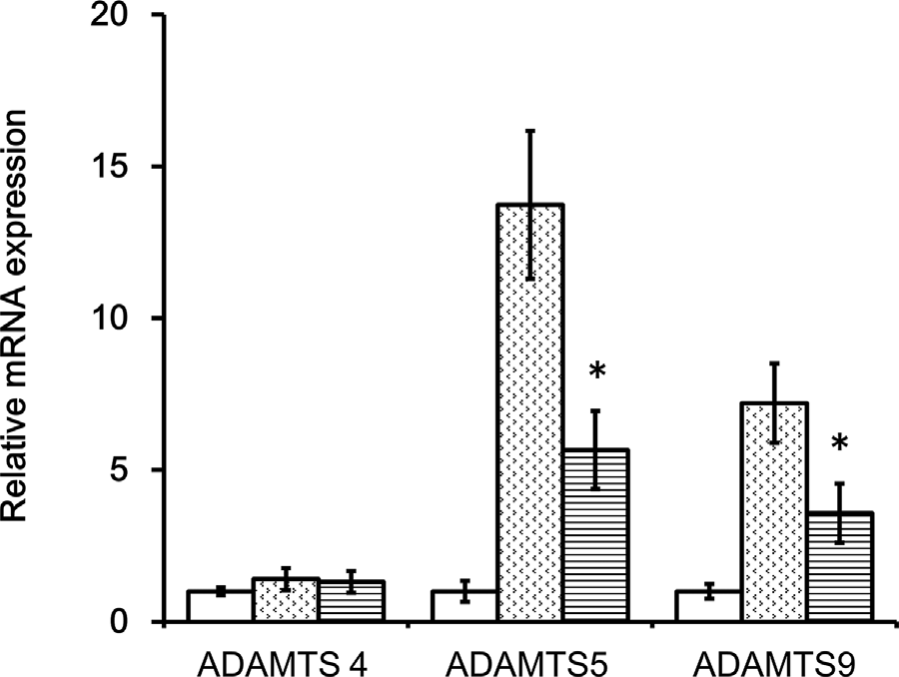
Intra‐articular HA injection attenuates *ADAMTS5* and *ADAMTS9* mRNA expressions in a rat OA model. HA was injected twice (days 5 and 7 post‐surgery) into the knee joint and mRNA levels were determined on day 10. *ADAMTS4*, *5*, and *9* transcript levels were compared between rats with and without HA2700 injection (*n* = 10 per group). The expression level of mRNA in sham‐operated rat was indicated as 1.0 and relative mRNA expression level in each OA rats was indicated as—fold change. Open bars, sham operation; dot bars, OA operation with PBS‐injection; horizontal stripe bars, OA operation with HA‐injection. *p < 0.05 versus PBS‐injected rats. Please note that the levels of *ADAMTS5* and *9* were suppressed by intra‐articular HA injection.

We assessed the effect of HA on *Adamts* mRNA expression by qRT‐PCR in a rat OA model by injecting 50 µl HA2700 (10 mg/ml) or PBS as a control into the knee joint of OA rats 5 and 7 days after surgery (Fig. S‐5), with mRNA extracted on day 10. We found that the levels of *Adamts5* and *9*, but not *Adamt4*, were increase in rat OA and these increased *Adamts5* and *9* mRNAs were attenuated by HA injection (Fig. 4).

### HA Protects Cartilage Against Destruction in OA

**Figure 5:**
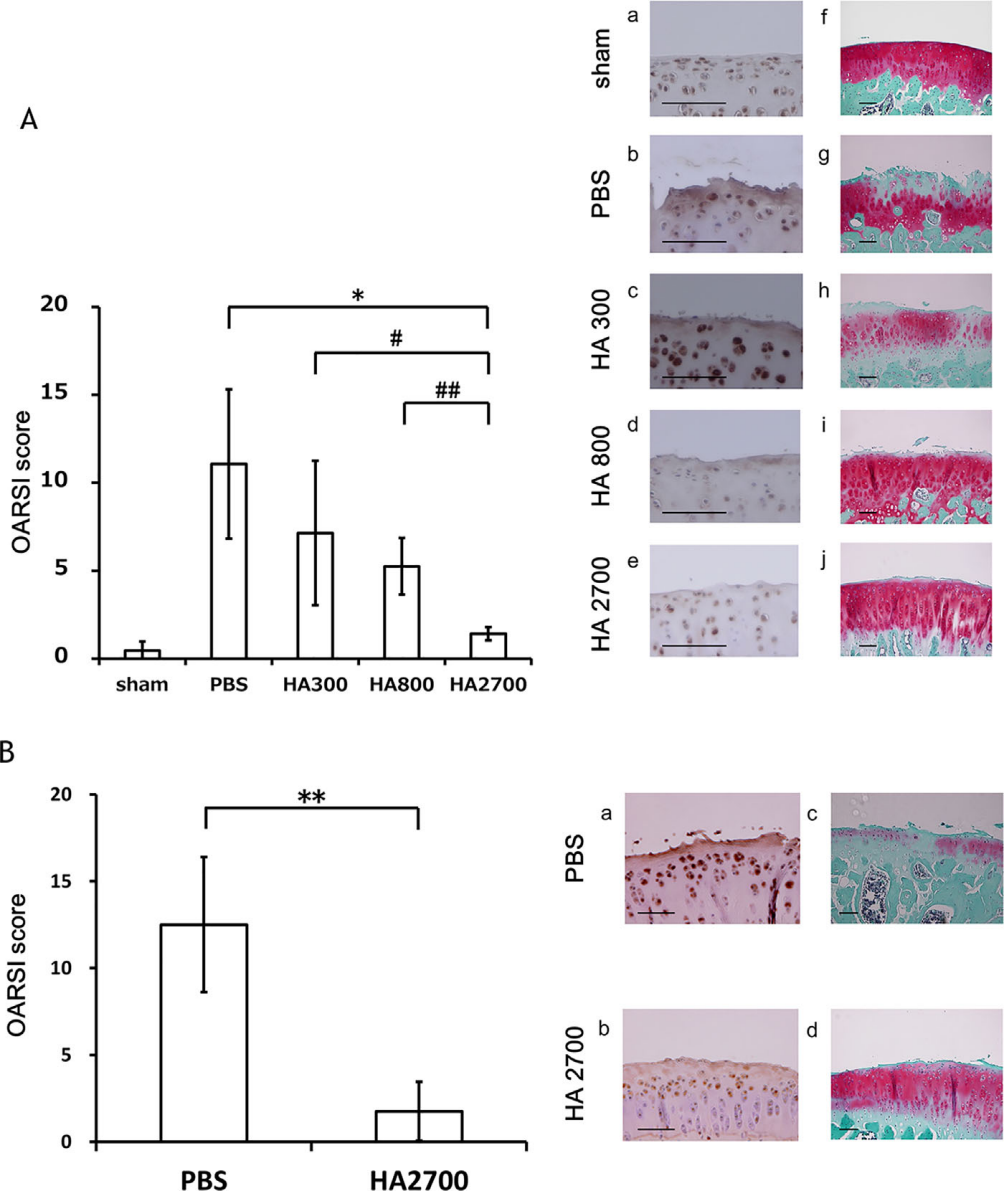
Intra‐articular HA injection protects against knee cartilage destruction in a rat OA model. (A) Influence of HA size. Rats were divided into five treatment groups (sham, PBS, HA300, HA800, and HA2700: *n* = 10 per group). The left panel shows OARSI scores 6 weeks post‐surgery. *p < 0.01 versus PBS‐injected rats; ^#^ and ^##^ *p* < 0.05 versus other HA‐injected rats. OARSI scores were lower in rats receiving intra‐articular HA injection; the effect was HA size‐dependent. Effects of intra‐articular HA injection (twice weekly for 6 weeks) were examined by immunohistochemistry using an anti‐NITEGE antibody (a–e) and by Safranin O staining (f–j). (B) The left panel shows OARSI scores 6 weeks post‐surgery. **p < 0.01 versus PBS‐injected rats. OARSI scores with short‐term intra‐articular HA injection (from days 5 to 19 after surgery) were lower as compared with that of PBS‐injected cartilage (*n* = 10 per group). Please note that the impact of short‐term HA injection (five injections within 19 days post‐surgery) was comparable with that of long‐term HA injection (eleven injections within 6 weeks) in rat OA cartilages as measured by OARSI scoring system. The cartilage destruction by ADAMTS was examined by immunohistochemistry using an anti‐NITEGE antibody (a and b) and by Safranin O staining (c and d). Scale bar, 30 µm.

We evaluated whether HA injection has a protective effect on cartilage in the rat OA model (Fig. S‐6). Intra‐articular injection of HA attenuated cartilage destruction 6 weeks after surgery, whereas OARSI scores were unchanged in the PBS‐injected group. HA2700 had a greater effect on the scores than HA300 or HA800 (Fig. 5A, left). We also examined aggrecan cleavage by immunohistochemistry and found that NITEGE immunoreactivity in knee joints was reduced by HA as compared to PBS injection. This effect was most apparent in rats treated with HA2700 as compared to HA300 or HA800 (Fig. 5A, right). HA injection did not influence rat body weight and health conditions (Table S‐1).

We also applied a different protocol for HA injection to determine whether short‐term HA intra‐articular injection can protect against cartilage destruction, since the induction of ADAMTS expression in our OA model was limited in the first 3 weeks after surgery. We initiated HA2700 injections 5 days after knee surgery; 50 µl HA 2700 (10 mg/ml) were injected five times every other day (Fig. S‐7), and knee joints were examined at week 6. As shown in Fig. 5B, cartilage destruction in femoral condyles was drastically reduced in HA‐ as compared to PBS‐injected rats. The OARSI score was almost similar between short‐term (i.e., five injections) and regular HA2700 injection after 6 weeks of surgery in our OA model.

## DISCUSSION

In this study, we examined the effects of HA in cultured chondrocytes/chondrocytic cells and in a rat OA model. We have previously found that IL‐1β and TNFα synergistically induced ADAMTS9.^24^ The qRT‐PCR Ct values for *ADAMTS9* without cytokine stimulation were smaller than those for *ADAMTS4* and *5* (Figs. 1A and 1B) in both types of cells. It means that *ADAMTS9* mRNA was much abundant without cytokine stimulation in these cells. The results of qRT‐PCR experiment revealed that Ct values for *ADAMTS9* in control rat were smaller than those for *ADAMTS4* and *5* in knee cartilage (Fig. 3B). Cartilage proteolytic activity of matrix degradative enzymes cannot be estimated only by its mRNA expression level, but by protein expression levels. Furthermore, it also depends on whether it is in the active form or inactive form. However, based on its high expression at 1–2 weeks after injury (Fig. 3B), ADAMTS9 could be primarily responsible for aggrecan degradation in the rat OA model.

The effect of HA was examined both in vivo and in vitro. The endotoxin concentration of HA used in our experiments was estimated to be under 0.00006 EU/ml. We therefore concluded that HA‐containing endotoxins did not influence matrix components or matrix degradative enzymes expression in this study. It should be noted that various receptors play roles in the molecular properties of HA. For instance, HA has been shown to inhibit *ADAMTS4* expression in human OA chondrocytes via CD44 and ICAM1.^23^ We previously reported that small HA molecules induced IL‐6 and monocyte chemoattractant protein‐1 (MCP‐1) expression in peripheral blood mononuclear cells in a dose‐ and time‐dependent manner.^32^ We found that HA‐increased CD44 expression, and treatment with an anti‐CD44 neutralizing antibody, reduced IL‐6, and MCP‐1 production in activated inflammatory cells, demonstrating that HA induces cellular response via CD44 in a size‐dependent manner.^32^ In addition, HA stimulates cell surface CD44 clustering in a size‐dependent manner.^33^ Washing out the HA pretreatment did not attenuate ADAMTS9 mRNA expression induced by inflammatory cytokines. This might suggest that effect of HA on this pathway requires the continuous presence of the exogenous HA in a concentration‐ and size‐dependent manner via CD44 cluster and HA bound to CD44 with a low‐affinity. HA binding to CD44 would up‐regulate expression of *type II collagen* via TGFβ1 signaling pathway.^34^ Expression of *Aggrecan* and *type II collagen* by HA2700 pretreatment alone (no cytokines) demonstrates that HA2700 can both interfere with cytokine‐mediated inhibition (presumably via TLR receptors) and stimulate matrix synthesis. Regarding HA receptor for endocytosis (HARE), there is another report demonstrating that low molecular weight HA (40–400 KDa) induced NF‐κB mediated gene activation and ERK1/2 activation via HARE, while high molecular HA did not.^35^ Therefore, HARE is excluded from our case. It was reported that KIAA1199 highly expressed in RA and OA synovial tissue. HA may attenuate cytokine‐induced KIAA1199 expression levels and KIAA1199 (also called Cell migration‐inducing and HA‐binding protein: CEMIP) may contribute to the effect of HA observed in this study.^36^ Based on these data, the greater activity of HA2700 might result from greater co‐operative binding (clustering) of HA receptors.

In our rat OA model, *ADAMTS5* and *9* but not *ADAMTS4* mRNA expression was increased in the operated knee. Aggrecan cleavage has been shown to increase following surgery and peak at week 4, followed by a decline at 6 weeks. Fig. 5A shows the considerable anti‐NITEGE reactivity was proximal to the cell surface. Interpretation of the anti‐NITEGE reactivity is difficult because the level of aggrecan neo‐epitope is not controlled by aggrecanase activity alone. The G1‐NITEGE species is also endocytosed by chondrocytes for lysosomal degradation,^37^ implying that an increased level of neo‐epitope reactivity can be due to an increased production, a decreased endocytosis, or both. Summerville et al., have reported that ADAMTS9 is secreted as mature form, is located near the cell surface, and is involved in aggrecan degradation.^38^ Our observation was in line with their concept of ADAMTS9, that is the fact that G‐NITEGE reactivity localization near the chondrocyte cell surface may indicate that ADAMTS9 had some unique role in OA onset or development. In rat OA model, we observed a substantial reduction in Safranin O staining corresponding to structural cartilage damage 4–6 weeks post‐surgery, which was mainly attributable to increased proteolytic activity by ADAMTS. Moreover, we found that *ADAMTS5* and *9* mRNA levels were transiently upregulated in rats with OA. ADAMTS5 deficiency had protective effects against cartilage loss,^39^, ^40^, ^41^ however, it was shown that deletion of ADAMTS5 in vivo did not prevent aggrecan degradation.^41^ They proposed that ADAMTS5 ablation may induce expression of other proteolytic enzymes or enzymatic activity (ADAMTS9 and others), or induce endocytotic clearance activity.^37^ Furthermore, it was recently reported that ADAMTS5 functions as a pro‐fibrotic and anti‐chondrogenic factor by limiting glucose uptake in cartilage degradation.^42^ Therefore, ADAMTS5 might have several unexpected functions in rodent OA, and ADAMTS9 may play a unique role in aggrecanolysis in OA development.

The significance of ADAMTS9 in OA was not clearly understood and further investigation is required to elucidate the role of ADAMTS9 on cartilage destruction in OA. Regarding ADAMTS4, it was not increased our in vivo model but many human OA studies have found that ADAMTS4 is upregulated in OA cartilage.^43^ This discrepancy may be due to inter‐species differences, particularly in relation to the period of disease development. In addition, we have recently found that the loss of ADAMTS4 resulted in the attenuation of both atherosclerotic plaque formation and the plaque stability.^44^ Our results suggest that HA tended to suppress the mRNA expression of more than one ADAMTS, implying that aggrecanase activity can be controlled by HA. It is interesting that our HA injection trial protected articular cartilage in surgical model. In fact, the in vivo effect was more prominent than in vitro results. This can be explained at least in part by the biophysical effect of HA. As HA adds viscoelasticity to the knee joint, the cartilage may be protected from mechanical stress. In addition, injected HA may affect other tissues including synovium. Although we examined the effect only in OUMS‐27, HA may affect other types of cells. The detailed mechanism of HA should be elucidated in the future study.

We also demonstrated short‐term intra‐articular HA2700 injection (i.e., five‐time injection) to rat OA model was enough to protect cartilage destruction after 6 weeks of surgery. As compared with the OARSI score in the long‐term HA‐injected cartilage, the OARSI score in the short‐term was comparable. It is interesting that short‐term HA injection was performed when ADAMTSs were increased in this rat model. It should be noted that HA injection was useful when expression of *ADAMTS5* and *ADAMTS9* were most activated in articular cartilage. That is, expression of these transcripts was transiently induced in our rat model and HA attenuated such induction, apparently resulting in protecting the cartilage from further destruction in this surgical model. Our observation is intriguing because mechanical instability caused by surgery was never fixed in our rat model. Our data also shed the light on the fact that attenuating ADAMTS induction by HA injection is important for protecting cartilage destruction from aggrecanase activity. We tested the effects of HA molecules of different sizes and found that their attenuation of cytokine‐induced *ADAMTSs* mRNA expression in OUMS‐27 cells was size dependent. HA2700 was previously reported to attenuate matrix metalloproteinase‐1, ‐3, and ‐13 levels in the synovial fluid of OA rabbits,^45^ which is in accordance with our findings. We also demonstrated a protective role for HA in experimental OA, as evidenced by the inhibition of ADAMTS‐mediated aggrecan cleavage, an effect that is exerted, in part, via downregulation of *ADAMTS* mRNA expression. In vitro, HA reduced inflammatory cytokine‐induced ADAMTSs and MMP mRNA expression by 10–30%. However, in rat OA model experiments (twice per week for 5 weeks, 5 shots, 1 shot), HA dramatically protected cartilage from destruction. It was estimated that injected HA was mostly released or depolymerized from mouse joints within 2 h.^46^ It was reported that HA penetrated more deeply in IL‐1β‐treated bovine articular cartilage.^47^, ^48^ Asari also reported that HA permeated well in the synovial tissue of a dog ACLT model.^49^ Injected HA penetrated into various tissues including the cartilage, synovial tissue, tendons, and ligaments. We anticipated that HA had biological functions (anti‐inflammation, inflammatory cytokines inducing the down‐regulation of matrix component attenuation), but HA protected cartilage even more effectively than expected from the in vitro analysis.

There were several limitations to this study. First, we chiefly used OUMS‐27 cells in our in vitro experiments. These cells are derived from a chondrosarcoma and are therefore not identical to normal chondrocytes. In our study, OUMS‐27 cells expressed chondrocyte‐specific ECM molecules such as aggrecan and type II collagen and exhibited similar aggrecan metabolism. Kunisada et al. first cloned this cell line and reported that OUMS‐27 expressed type I, II, and III collagen.^50^ Collagens and proteoglycans are the main structural components of OUMS‐27. Aggrecan cleavage by ADAMTS was also observed in OUMS‐27 cells (data not shown), suggesting that OUMS‐27 retains chondrocytic properties and can therefore serve as an in vitro model of cytokine‐induced aggrecanase. Therefore, we used this cell line to analyze the effects of HA on the ECM.

Second, the precise role of *ADAMTS9* in OA has yet to be elucidated. Because reliable anti‐ADAMTS9 antibody has never been reported to determine its protein localization in rats, we could not confirm the role of ADAMTS9 in early OA cartilage. In future studies, suitable anti‐ADAMTS9 antibody will clarify this question. Mice with cartilage‐specific deletion of *ADAMTS9* may also be useful for examining this, since global *ADAMTS9* knockout mouse is embryonically lethal. Our results also demonstrated that, in addition to its viscoelastic properties in cartilage, HA can inhibit multiple ADAMTSs (including ADAMTS9) in vitro and in vivo, thereby protecting against aggrecan degradation in OA.

Third, in the absence of cytokines 5 h treatment with HA affected matrix molecules such as aggrecan and type II collagen, while it did not affect ADAMTS9 mRNA expression (Fig. S‐3). As HA can bind its receptor, it may cause additional effects on aggrecan and collagen expression. Indeed, HA‐treated in vivo model was not able to completely protect cartilage destruction after 9 weeks (data not shown). Accordingly, we believe that HA showed the cartilage protective effect, mostly attenuating cartilage degrading enzymes.

In conclusion, HA attenuated the inflammatory cytokine‐induced expression of *ADAMTS* mRNAs in vitro and protected articular cartilage from degradation in an experimental OA model. These effects were dependent on the size of the HA molecule. HA protected cartilage through its biological functions. Our findings provide insight into the molecular basis of the beneficial effects of HA injections in the treatment of OA.

## AUTHORS' CONTRIBUTIONS

TO designed the study and wrote the initial draft of the manuscript. KA and JI contributed to RNA purification and RT‐PCR analysis. AS, KK, and MZC contributed to operating rat OA model surgery. OFH, TO, and KN contributed to cell culture and cytokines stimulation. IK contributed to preparing paraffin blocks and slice staining. SH contributed to cell culture, study design, and project supervision. All other authors have contributed to data collection and interpretation, and critically reviewed the manuscript. All the authors approved the final version of the manuscript and agree to be accountable for all aspects of the work in ensuring that questions related to the accuracy or integrity of any part of the work are appropriately investigated and resolved.

## Supporting information

Additional supporting information may be found online in the Supporting Information section at the end of the article.

**Figure S1**. HA suppresses ADAMTS9 mRNA expression in cytokine‐stimulated cells.
**Figure S2**.Influence of HA washing out after HA treatment on ADAMTS9 mRNA expression in OUMS‐27 cells.
**Figure S3**. Influence of HA pre‐treatment time on ADAMTS9 mRNA expression in OUMS‐27.
**Figure S4**. HA treatment restores aggrecan and collagen mRNA expression reduced by cytokine stimulation.
**Figure S5**. The time schedule for intra‐articular injection of HAThe five days after knee surgery, the first HA was administered and the next HA was intra‐articularly injected at day seven after surgery. Then, the tissue was taken at day ten after surgery for the analysis.
**Figure S6**. The time schedule for intra‐articular injection of HA The five days after knee surgery, the first HA was administered and HA was intra‐articularly injected twice a week until 40 days after surgery. Then, the tissue was taken at six weeks after surgery for the analysis.
**Figure S7**. The time schedule for intra‐articular injection of HAThe five days after knee surgery, the first HA was administered and HA was intra‐articularly injected twice a week until 19 days (five injections as a total) after surgery. Then, the tissue was taken at six weeks after surgery for the analysis.
**Table S1**. The effects of intra‐articular injection of HA (HA300, HA800 and HA2700) on OA model rats. The weight of each group was measured from operated day to 6th week. Values represent mean ± S.D. (n = 10 per group).
