# Supplementary material for: High molecular weight hyaluronan protects cartilage from degradation by inhibiting aggrecanase expression
Source: J Orthop Res. 2018 Sep 7;36(12):3247–55. doi: 10.1002/jor.24126 (PMC6585799; doi:10.1002/jor.24126)
Supplement: Supplementary file 1 — Figure S1. HA suppresses ADAMTS9 mRNA expression in cytokine‐stimulated cells. Figure S2.Influence of HA washing out after HA treatment on ADAMTS9 mRNA expression in OUMS‐27 cells. Figure S3. Influence of HA pre‐treatment time on ADAMTS9 mRNA expression in OUMS‐27. Figure S4. HA treatment restores aggrecan and collagen mRNA expression reduced by cytokine stimulation. Figure S5. The time schedule for intra‐articular injection of HAThe five days after knee surgery, the first HA was administered and the next HA was intra‐articularly injected at day seven after surgery. Then, the tissue was taken at day ten after surgery for the analysis. Figure S6. The time schedule for intra‐articular injection of HA The five days after knee surgery, the first HA was administered and HA was intra‐articularly injected twice a week until 40 days after surgery. Then, the tissue was taken at six weeks after surgery for the analysis. Figure S7. The time schedule for intra‐articular injection of HAThe five days after knee surgery, the first HA was administered and HA was intra‐articularly injected twice a week until 19 days (five injections as a total) after surgery. Then, the tissue was taken at six weeks after surgery for the analysis. Table S1. The effects of intra‐articular injection of HA (HA300, HA800 and HA2700) on OA model rats. The weight of each group was measured from operated day to 6th week. Values represent mean ± S.D. (n = 10 per group). [file JOR-36-3247-s001.doc]

**Supplement Information**

**High molecular weight Hyaluronan protects cartilage from degradation by inhibiting Aggrecanase expression**

Takashi Ohtsuki, Keiichi Asano, Junko Inagaki, Akira Shinaoka, Kanae Kumagishi-Shinaoka, Mehmet Zeynel Cilek, Omer Faruk Hatipoglu, Toshitaka Oohashi, Keiichiro Nishida, Issei Komatsubara, Satoshi Hirohata

**Supplemental Figures and Table**

**Supplementary Figure S-1.** **HA suppresses *ADAMTS9* mRNA expression in cytokine-stimulated cells**

(A) Influence of HA size on ADAMTS9 mRNA expression in chondrocytic cells. OUMS-27 cells were pretreated with 1.0 mg/ml HA300, HA800, or HA2700 for 5 h, then stimulated with 10 ng/ml IL-1β and TNFα containing 0% FBS DMEM for 6 h. *ADAMTS9* transcript level was determined relative to the level in unstimulated control cells by qRT-PCR. Values represent mean ± S.D. (n = 6 per group). *P < 0.05 vs. cytokine-stimulated cells without HA pretreatment. ^#^P < 0.05 vs. cytokine-stimulated cells with HA800 pretreatment. (B) Influence of HA size on ADAMTS9 mRNA expression in NHAC-kn cells. NHAC-kn cells were pretreated with 1.0 mg/ml HA300, HA800, or HA2700 for 5 h, then stimulated with 10 ng/ml IL-1β and TNFα for 6 h. *ADAMTS9* transcript level was determined relative to the level in unstimulated control cells by qRT-PCR. Values represent mean ± S.D. (n = 6 per group). *P < 0.05 vs. cytokine-stimulated cells without HA pretreatment. ^#^P < 0.05 vs. cytokine-stimulated cells with HA800 pretreatment. (C) Influence of HA concentration on *ADAMTS9* mRNA expression in OUMS-27. OUMS-27 cells were pretreated with HA2700 for 5 h, then stimulated with IL-1β and TNFα for 6 h. *ADAMTS9* transcript level was determined relative to the level in unstimulated control cells by qRT-PCR. Values represent mean ± S.D. (n = 6 per group). *P < 0.05 vs. cytokine-stimulated cells without HA pretreatment.

**Supplementary Figure S-2. Influence of HA washing out after HA treatment on ADAMTS9 mRNA expression in OUMS-27 cells**

OUMS-27 cells were pretreated with 1.0 mg/ml HA2700 for 5 h, then washed out HA by PBS twice and stimulated with 10 ng/ml IL-1β and TNFα for 6 h. The level of each mRNA transcript was determined relative to the level in unstimulated control cells by qRT-PCR. Values represent mean ± S.D. (n = 6 per group). *P < 0.05 vs. cytokine-stimulated cells without HA pretreatment.

**Supplementary Figure S-3. Influence of HA pre-treatment time on *ADAMTS9* mRNA expression in OUMS-27.**

OUMS-27 cells were pretreated with HA2700 for 1, 3 and 5 h, then stimulated with IL-1β and TNFα for 6 h. *ADAMTS9* transcript level was determined relative to the level in unstimulated control cells by qRT-PCR. Values represent mean ± S.D. (n = 6 per group). *P < 0.05 vs. cytokine-stimulated cells without HA pretreatment.

**Supplementary Figure S-4.** **HA treatment restores *aggrecan* and *collagen* mRNA expression reduced by cytokine stimulation**

OUMS-27 and NHAC-kn cells were treated for 0–48 h with 10 ng/ml IL-1β and TNFα. *Aggrecan* (A) and *type II collagen* (B) mRNA expression relative to levels in unstimulated cells was determined by qRT-PCR. Values represent mean ± S.D. (n = 6 per group). *P < 0.05 vs. unstimulated cells. *Aggrecan* (C) and *type II collagen* (D) mRNA expression in NHAC-kn cells relative to levels in unstimulated cells was determined by qRT-PCR. Values represent mean ± S.D. (n = 6 per group). *P < 0.05 vs. unstimulated cells. (E, F) OUMS-27 cells were pretreated with 1.0 mg/ml HA300, HA800, or HA2700 for 5 h, then stimulated with IL-1β and TNFα for 24 h; *aggrecan* (E) and *type II collagen* (F) transcript levels were determined relative to levels in unstimulated control cells by qRT-PCR. Values represent mean ± S.D. (n = 6 per experimental group). #P < 0.05 vs. unstimulated cells. *P < 0.05 vs. cytokine-stimulated cells without HA pretreatment. **P < 0.01 vs. cytokine-stimulated cells without HA pretreatment.

**Supplementary Figure S-5. The time schedule for intra-articular injection of HA**

The five days after knee surgery, the first HA was administered and the next HA was intra-articularly injected at day seven after surgery. Then, the tissue was taken at day ten after surgery for the analysis.

**Supplementary Figure S-6. The time schedule for intra-articular injection of HA**

The five days after knee surgery, the first HA was administered and HA was intra-articularly injected twice a week until 40 days after surgery. Then, the tissue was taken at six weeks after surgery for the analysis.

**Supplementary Figure S-7.** **The time schedule for intra-articular injection of HA**

The five days after knee surgery, the first HA was administered and HA was intra-articularly injected twice a week until 19 days (five injections as a total) after surgery. Then, the tissue was taken at six weeks after surgery for the analysis.

**Supplementary Table S-1.** The effects of intra-articular injection of HA (HA300, HA800 and HA2700) on OA model rats. The weight of each group was measured from operated day to 6^th^ week. Values represent mean ± S.D. (n = 10 per group).
